# Using multielectrode arrays to investigate neurodegenerative effects of the amyloid-beta peptide

**DOI:** 10.1186/s42234-021-00078-4

**Published:** 2021-10-28

**Authors:** Steven Schulte, Manuela Gries, Anne Christmann, Karl-Herbert Schäfer

**Affiliations:** 1 Department of Informatics and Microsystems and Technology, University of Applied Science Kaiserslautern, 66482 Zweibrücken, Germany; 2 Department of Pediatric Surgery, Medical Faculty Mannheim, University of Heidelberg, 68167 Mannheim, Germany

**Keywords:** Neurodegenerative diseases, Alzheimer’s disease, Multielectrode arrays, Pharmacology, Target identification, Drug discovery, Neural circuit activity

## Abstract

**Background:**

Multielectrode arrays are widely used to analyze the effects of potentially toxic compounds, as well as to evaluate neuroprotective agents upon the activity of neural networks in short- and long-term cultures. Multielectrode arrays provide a way of non-destructive analysis of spontaneous and evoked neuronal activity, allowing to model neurodegenerative diseases *in vitro*. Here, we provide an overview on how these devices are currently used in research on the amyloid-β peptide and its role in Alzheimer’s disease, the most common neurodegenerative disorder.

**Main body:**

Most of the studies analysed here indicate fast responses of neuronal cultures towards aggregated forms of amyloid-β, leading to increases of spike frequency and impairments of long-term potentiation. This in turn suggests that this peptide might play a crucial role in causing the typical neuronal dysfunction observed in patients with Alzheimer’s disease.

**Conclusions:**

Although the number of studies using multielectrode arrays to examine the effect of the amyloid-β peptide onto neural cultures or whole compartments is currently limited, they still show how this technique can be used to not only investigate the interneuronal communication in neural networks, but also making it possible to examine the effects onto synaptic currents. This makes multielectrode arrays a powerful tool in future research on neurodegenerative diseases.

## Background

Neurodegenerative diseases are hallmarked by a massive, pathological death of neurons, which leads to a decline in cognitive and/or motoric abilities. Symptoms depend on the neural compartment that is impacted and its function. The most common neurodegenerative disorder, Alzheimer’s disease (AD), is characterized by a loss of neurons in certain cerebral cortical regions, including hippocampus and temporoparietal cortex (St George-Hyslop and Petit 2005), followed by cognitive disturbances. Based on previous studies to develop potential treatments of neurodegenerative diseases and the etiological mechanisms involved, it has become a crucial task to understand the role of possibly harmful peptides in processes leading to neuronal cell death. Given the electrogenic features of neural tissue, studying the impact of such endogenous neurotoxic substances on neuronal function is an absolute demanding goal. So far, investigations on the impact of these molecules have been based largely on electrophysiological experiments. Loss of electrical functionality indicates an impairment of compromised neurons and is seen as the initiation point of the typical symptoms in neurodegenerative disorders. To reveal the influence of potentially neuropathogenic peptides such as amyloid-β (Aβ), whose aggregated form might be the culprit in AD-related neurodegeneration, its direct effect upon single neurons as well as on whole neural networks needs to be evaluated. For these purposes, different methods have emerged during the last decades and to choose an appropriate one largely depends on the scope of analysis. For single-cell examination, the patch clamp technique is a widely used tool, allowing to record intracellular voltages of single neurons using sharp glass pipettes, which restricts measurements to a few neurons per experiment. Although multi-cell approaches have been developed (Wagner et al. 2015), these recordings can be time and effort consuming.

To deduct signals of multiple neurons or whole neural networks simultaneously, multielectrode arrays (MEAs) have been used for many decades. MEAs allow to investigate the impact of neurotoxic compounds upon complex neuronal networks *in vitro* and in specific cases even *in vivo* (Wood et al. 2004). The MEA technology is based on extracellular recordings and on the performance of mainly metal electrodes which are arranged in large arrays. In contrast to patch clamp techniques, these devices provide a non-invasive method to record electrical signals from whole neuronal networks and, by increasing the number of electrodes in the array, allow to magnify the scope to single neurons. Moreover, individual approaches also allow to stimulate the networks with one of the recording electrodes.

Although there are several other electrophysiological methods which can be used to elucidate the effect of pharmacologically active substances onto neural networks *in vitro*, like calcium imaging, MEA technique has some key features reviewed here that make it a powerful tool in the analysis of neurodegenerative diseases. This review tries to provide a short overview of the potential use of MEAs in the evaluation of neuropathological effects of Aβ. First, there will be a short description of MEA technology and its basics followed by a state of the art of AD research using the MEA technology. Finally, current work in the field of AD research using MEAs is presented.

## Main text

### MEA technology and applications

**Fig. 1:**
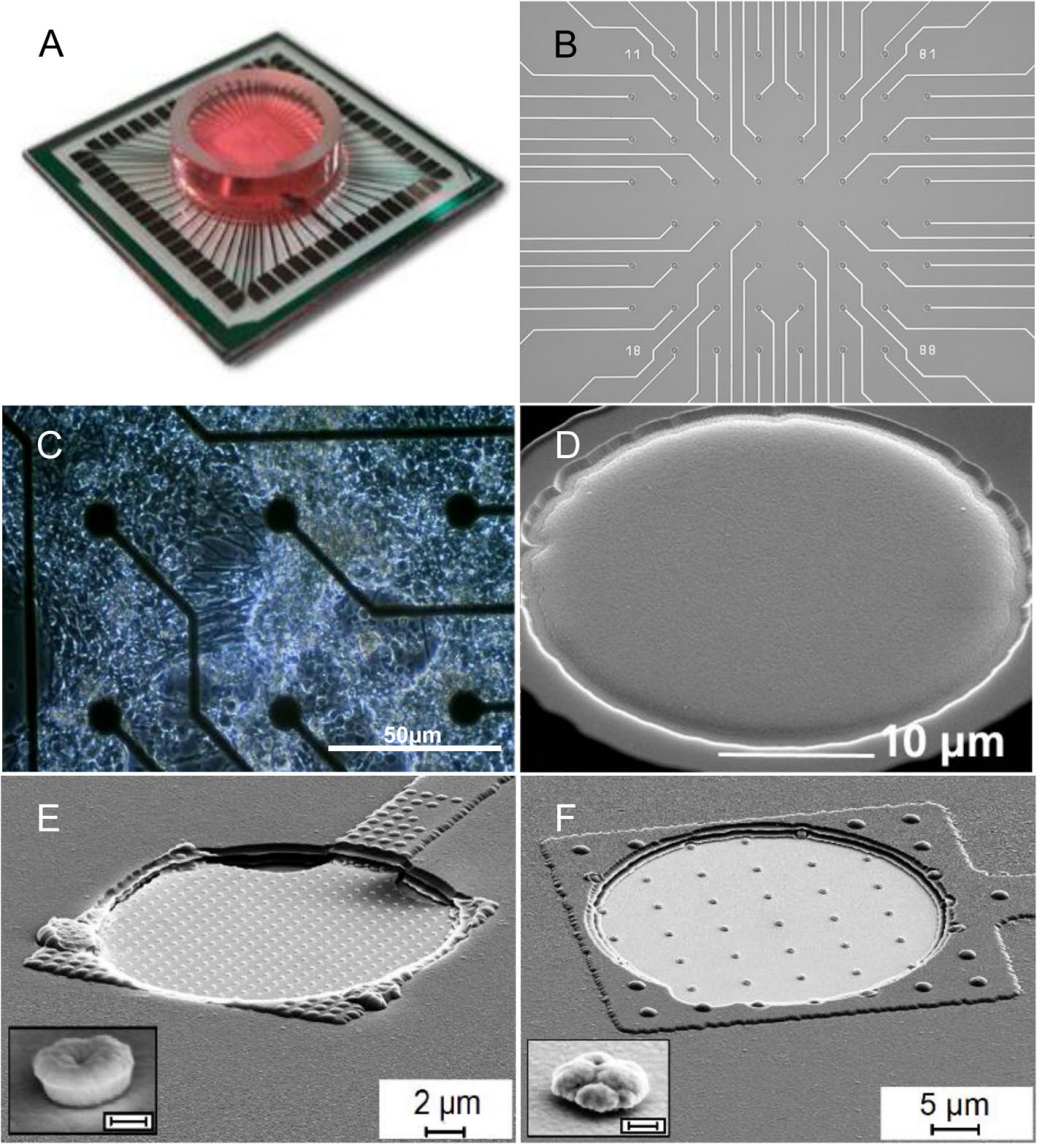
Exemplary pictures of MEA chips and electrodes of different shapes. **A** multielectrode array from Multichannel Systems© with 60 electrodes and chamber containing cell culture medium. **B** Arrangement of electrodes in an array of 60. **C** Clusters of myenteric neurons growing on planar microelectrodes, scale bar 50 μm. **D** SEM picture of a planar electrode, scale bar 10 μm. **E** and **F** SEM pictures of nanostructured MEA electrodes with tube- and mushroom-like nanostructures, scale bars 2 and 5 μm, respectively (insets: magnification of nanostructures, scale bar: 200 nm; taken with permission from Decker et al., 2019)

MEAs are substrate-integrated arrays of ten to thousands of metal electrode contacts (Fig. 1 A and B) and offer the possibility to record neuronal activity *in vitro* (Fig. 1 C) as well as *in vivo*, non-invasively and with high spatial and temporal resolution (Ness et al. 2015). The number of electrodes is a key feature enhancing spatial resolution in particular, revealing the main advantage over e.g. EEG recordings (Obien et al. 2015). The shape of the electrodes can vary from planar (Fig. 1D) to 3-dimensional (Fig. 1E and F; (Decker et al. 2019)) and even mimicking the structure of the natural anchor proteins of the extracellular matrix, such as collagen, to improve cell coupling and adhesion (Nowduri et al. 2020). The MEA technique allows to record local field potentials (LFP) and extracellular action potentials (EAPs) simultaneously. In specific cases, using the 3D electrode *in vitro* approach, a deduction of subcellular activity (e.g. from dendrites, somata and axons) can be accomplished (Spira and Hai 2013). Moreover, long-term experiments that include the implementation of convective perfusion for automated culturing of organotypic slices, open interesting perspectives to follow the impact of individual compounds along prolonged time axes (Killian et al. 2016). EAPs are defined as action potentials recorded by electrodes placed in the extracellular space, in contrast to intracellular action potentials (IAPs) obtained in patch clamp measurements. Neuronal EAPs usually are around tens to hundreds of microvolts in amplitude and less than 2 milliseconds (ms) in duration (Buzsáki et al. 2012). MEA microelectrodes detect the changes in the extracellular field which are caused by current flows of ionic processes of the neurons closest to the electrode, while this is not restricted to neuronal cells (Quiroga et al. 2013). This electrical field is usually referred to as the LFP and resembles a superposition of all ionic processes (Herreras 2016). Thus, any neuronal transmembrane current contributes to the potential detected on a microelectrode, while the characteristics of this potential resemble the sum of every neuronal source in the vicinity of this particular electrode (Buzsáki et al. 2012). Hence, the EAP of many neurons can be the source of the LFP, though it is still unclear how EAPs contribute to the extracellular field in detail (Obien et al. 2015).

**Fig. 2:**
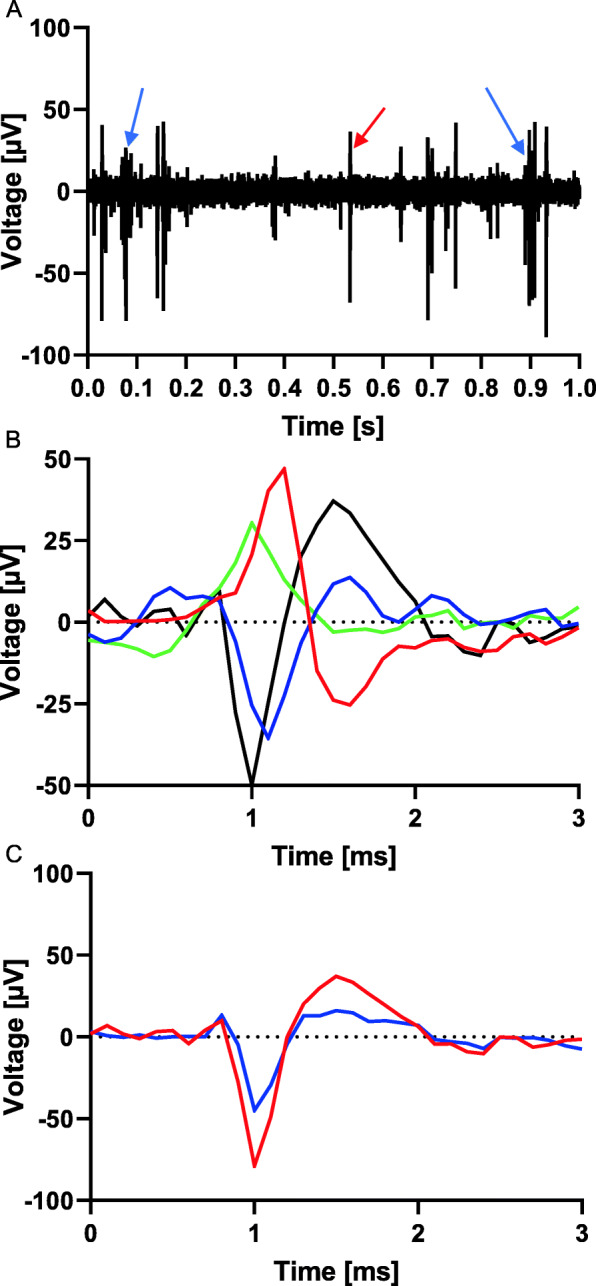
Illustration of signals recorded with multielectrode arrays. **A** One second stream of recording from a single electrode, including single spikes (red arrow) and bursts (blue arrows). **B** Overlay of single spikes of different waveforms, which suggest different neuronal subtypes as the source. **c** Overlay of two spikes with different amplitudes but similar waveforms, either indicating different neuronal subtypes or distances of the respective spiking neurons from the recording electrode

Action potentials measured at an electrode are commonly referred to as “spikes”, which in the context of extracellular recordings are characterized as voltage signals that exceed a certain threshold; if several spikes occur in succession with an interspike interval of less than 3ms, they are commonly referred to as “bursts” (Fig. 2 A). These signals exhibit certain features like waveform, amplitude and frequency, all of which depend on neuronal subtype, distance from the electrode and stimulus (Fig. 2B and C). This makes feature extraction and spike sorting an essential part in the analysis of these recordings, to understand which kinds of neurons take part in the communication within a neural network (Lewicki 1998).

This “crosstalk” is often recorded spontaneously, that is, without any application of external stimuli, to create a baseline which can serve as a reference. On the other hand, these responses can also be evoked, either by applying substances with a known or yet to be tested effect on neural tissue, by electrically stimulating cells through the integrated electrodes (Hales et al. 2012), or a combination of both. In this regard, an interesting approach to understand the ongoing processes within the neuronal networks, or to identify role and type of neurons involved, is the pharmacological blockage of individual subtypes with subsequent analysis of the impact on spike formation (Gramowski et al. 2006).

Taken together, these modifications can bring the experimental environment closer to the *in vivo* situation, making *in vitro* MEA recordings a powerful tool in pharmacological and neurophysiological research. Many studies have delivered insight on how to use these devices i.e. for drug-screening, in order to estimate neurotoxicity and overall influence of substances onto isolated compartments of the nervous system (Morefield et al. 2000; Gopal et al. 2011; Gramowski et al. 2011), or even to use neurons as biosensors for a wide range of chemicals using methods of genetical engineering (Patriarchi et al. 2018; Sabatini and Tian 2020).

However, the application of MEAs extends beyond recordings of isolated neural networks *in vitro* to the implantation of electrodes into cerebral tissue of animals and humans, where again the summation of signals of many sources is recorded. This provides an even closer look into the *in vivo* situation but also makes analyses like spike sorting a more complex task, due to the drastically increased number of sources that give rise to the signals at a particular electrode. In this context, new approaches of data processing have emerged out of the ongoing progress of artificial intelligence, from methods of Deep Learning in particular. Here, artificial neural networks are trained to recognize spikes using large sets of simulated signals (see (Tavanaei et al. 2019) for a review of current concepts). These programs are not only able to detect spikes in an unsupervised fashion, but can also perform tasks like spike sorting (Saif-Ur-Rehman et al. 2019) and predicting the distance of neurons from the recording electrode (Buccino et al. 2018).

Nevertheless, there are several advantages of *in vitro* MEA recordings of dissociated neurons over *in vivo* approaches and acute brain slice recordings, the most pragmatic being the fact that cultures and recordings can be sustained for a prolonged period (Potter and Demarse 2001). This in turn opens up the opportunity to study the formation and development of the geometry and electrical activity of neural networks (Shahaf and Marom 2001; Hofmann and Bading 2006). In addition, temporal resolution of *in vitro* MEA recordings can outrun techniques for imaging neuroelectrical activity, like 2-photon calcium imaging (Delgado Ruz and Schultz 2014). Considering these advantages, together with the possibility to study the effect of pharmacologically active peptides, recordings of cultured neurons on MEAs may facilitate the study of neurodegenerative disorders like AD (Jones et al. 2011).

### Alzheimer’s disease

AD is mainly characterized by a loss of neurons in distinct areas of the brain (i.e., hippocampus and temporoparietal neocortices). Most research on therapeutics has focused on the histopathological hallmarks, cortical amyloid plaques and neurofibrillary tangles, in association with their most insoluble components, the Aβ peptide (Glenner, G. and Wong 1984) and the microtubule-associated protein tau (Wood et al. 1986), respectively. In this context, aggregates termed senile or amyloid plaques are often seen as the culprit in AD pathophysiology and are another prominent neuropathological feature. These aggregates are complex, extracellular, fibrillar deposits of several proteins, while the main component is the neurotoxic Aβ_42_ peptide, a 42 amino acids long molecule derived from the β-amyloid precursor protein βAPP (St George-Hyslop and Petit 2005). In this review, we will focus on studies that analyzed the effect of Aβ on to neural networks using *in vitro* MEA approaches, as different isoforms of Aβ are present in all of the plaques that are linked to ‘normal’ aging and Alzheimer’s disease, regardless of size, shape, aggregation state, location, or overall composition (Walker 2020).

There are several propositions that have been postulated to explain cognitive dysfunctions accompanying this neurodegenerative disease on the neurophysiological level. The current understanding is that these damages are most probably not directly originated in neuronal apoptosis, at least not in earlier stages of AD. Aβ_42_ is known to bind especially to the α7 subunit of the nicotinic acetylcholine receptor (nAChR), at least in the central nervous system (Wang et al. 2000). This might explain why cholinergic neurons seem to be the group of cells that are affected first by the disease.

Multiple studies have delivered evidence indicating that oligomers of Aβ also have a direct effect on synapses and that the typical symptoms of AD are derived from an impaired synaptic function (Townsend et al. 2006; H. Alzoubi et al. 2011; Koffie et al. 2011). This also results in changes in synaptic plasticity, a factor commonly described as the ability of neural tissue to form new synaptic connections and enhance existing ones as a part of memory and learning. To investigate on this particular property, long-term potentiation (LTP) is often taken to model synaptic plasticity, and the MEA-technology is a valuable tool to measure these events, normally with conventional MEAs, but also with especially designed electrode arrays where hippocampal slices can be placed appropriately on the MEA, so that the Schaffer collaterals can easily be addressed (Zheng et al. 2021). LTP can be described as an enhancement of synaptic activity following short, high-frequency stimulation through afferent connections (Chen et al. 2000). This electrical activation, which can be simulated *in vitro* by applying voltages of certain frequencies via integrated electrodes, can evoke a particular kind of signals known as excitatory post-synaptic potentials (EPSPs), which are enabled through activation of voltage-gated neuronal dendrites (Johnston et al. 1996). A suppression of LTP in hippocampal tissue by monomeric and oligomeric Aβ_42_, respectively, could be found in several studies, suggesting a deleterious impact of Aβ_42_ on synaptic plasticity (Chen et al. 2000; Wang et al. 2002).

Besides these findings, there seems to be a rise in excitability of neurons in the progress of AD. This hyperexcitability may be linked to changes in the geometry of dendrites, which can lead to alterations of their electrical properties (Spruston 2008). Indeed, reduction of dendritic branching and length are found in hippocampal neurons of patients with AD (Grutzendler et al. 2007), which may render a neuron electrically more compact. This in turn could increase the efficiency with which synaptic currents are translated into postsynaptic and axosomatic depolarization, which would then raise action potential output (Johnston et al. 1996). Another consequence thereof might be abnormal circuit synchronization, which has been found to contribute to cognitive dysfunction in patients with AD (Minkeviciene et al. 2009).

### Revealing amyloid-β toxicity in multielectrode array experiments

During the last decade, several studies have investigated the relationship of aggregated Aβ onto neural networks grown on MEAs. Most of this research has been concentrated onto hippocampal neurons, as this cerebral area seems to be the first affected by the disease, leading to typical early symptoms of dementia. Cultures are either integrated as whole slices of hippocampal tissue or as dissociated cells. While the number of studies using the MEA technology to examine the impact of monomeric and aggregated species of Aβ onto neural tissue *in vitro* is comparatively low, they nevertheless show the power of this technique regarding the simulation of the *in vivo* situation, mimicking the communication and communication deficits between different cerebral compartments by electrical and chemical evocation of interneuronal signaling.

The changes in this communication elicited by Aβ include frequency and amplitude of spikes not only from neuronal, but also synaptic sources. Varghese and coworkers showed significant changes in firing rates of hippocampal neurons in rats following long-term application of different concentrations of oligomeric Aβ_42_ (Varghese et al. 2010) ranging between 100nM and 20µM. This study showed a dose-dependent relationship between Aβ_42_ concentration and the duration for reaching total abolishment of spiking activity. While all used concentrations of Aβ_42_ oligomers increased spiking activity over the course of a few hours, firing rates were depleted shortly thereafter. Increasing concentration not only led to earlier attenuation of electrophysiological function, but also to significant neural cell death. Furthermore, this effect could be partially reversed by co-culturing hippocampal neurons with the anti-amyloidogenic natural compound curcumin, which prevented firing rates from declining. Curcumin is a phenolic yellow pigment derived from the curry spice mixture that has been shown to have potent anti-inflammatory and antioxidant activities, and binds to species of Aβ (Yang et al. 2005).

The initial increase of spiking rates found here was also recently observed in another study (Henderson et al. 2019). There, the authors ascribe this feature to a hyperexcitability of hippocampal neurons following the application of Aβ_42_ oligomers over a course of 6 h, which was formerly described in a paper examining an APP mouse model of AD (Šišková et al. 2014).

The neuroprotective abilities of curcumin in the presence of Aβ_42_ oligomers were corroborated by Hoppe et al., which showed that curcumin prevents aggregated species of Aβ from decreasing total LFP of hippocampal neurons in a long-term measurement of 24 h (Hoppe et al. 2013). The authors propose that curcumin decreases Aβ_42_ induced attenuation of synaptically propagated neuronal activity. Several studies have put the scope of their MEA-based experiments to signals derived from synaptic currents and the effect of Aβ oligomers onto it. As it has been shown in the past, the cognitive dysfunction observed in AD patients may be caused by an impairment of synaptic transmission, at least in earlier stages, rather than neuronal apoptosis.

In these experiments, the analysis is focused on particular signals that are known to be of synaptic origin, which can either be accomplished by electrical stimulation via the integrated electrodes of MEAs or via usage of chemicals that antagonize synaptic potentials. Ahuja et al. exposed hippocampal cultures integrated into MEA chips to either theta-burst (TBS, 10 trains of 4 pulses with a total duration of 2 s) or high-frequency stimulation (HFS, two 1-s trains), both using a frequency of 100 Hz, to induce initial LTP (Ahuja et al. 2007), which was calculated as the average of evoked EPSPs in a period of 5 min. Following long-term exposure (24 h) to aggregated Aβ_42_ (1µM), they found no changes in spontaneous EPSPs, while the ability of hippocampal tissue to induce LTP was decreased globally. The same was found in experiments with mice of an AD model overexpressing Aβ_42_ and tau in combination (Chong et al. 2011).

Using a different approach in confirming the synaptic origin of measured signals, further studies not only showed declining of synaptic activity in hippocampal neurons following application of different concentrations of oligomeric Aβ_42_ (10nM, 1µM and 5µM), but also that activity returns to baseline after 120 h, indicating metabolic processing of oligomers (internalization and/or proteolysis) by hippocampal tissue (Lee et al. 2013). The synaptic origin of the signals was not ascertained recording them after electrical stimulation, but by inhibiting excitatory and inhibitory synaptic potentials using a mixture of certain antagonists, as described earlier (Serra et al. 2010). The addition of iron to hippocampal cultures seems to synergistically enhance the inhibition of synaptic signaling to an extent at which even subcytotoxic levels of Aβ suffice, as shown in a consecutive study (Shea 2014). Moreover, this effect could be reversed by adding zinc, which prevented frequency of synaptic signals from declining. Based on earlier studies on levels of iron and zinc in spinal fluid of individuals with AD versus healthy controls, the authors propose that the interaction of certain metals with physiological species of Aβ might be an initiation point of the disease, which so far has not been taken into consideration due to the relatively subtle impact on synaptic signaling, a factor that is certainly more difficult to examine in humans.

## Conclusions

Overall, the studies described here corroborate a state-of-the-art hypothesis on Aβ inducing neurotoxicity on an electrophysiological level using MEA technology. This technique opens a large field of experimental possibilities, ranging from pharmacological studies to the investigation of communication in neural networks, including whole brain slices and single cell type cultures. Hence, MEA technology is a highly suitable tool for the examination of neurodegenerative disorders such as AD, enabling the analysis of deleterious influences of disease-related peptides onto neural tissue not only on the level of the whole network, but also making it possible to examine the effects onto synaptic currents. This review focused on experiments studying the impact of Aβ onto hippocampal neurons *in vitro*, while current and future studies will also be based on multielectrode array measurements *in vivo*, using electrodes implanted into cerebral tissue, moving the scope even closer to the conditions that cause neurodegeneration and how the typical symptoms of these disorders emerge.

## Data Availability

Not applicable.

## Abbreviations

AD: Alzheimer’s disease
Aβ: amyloid-β
EAP: extracellular action potential
EPSP: excitatory post-synaptic potential
HFS: high-frequency stimulation
IAP: intracellular action potential
LFP: local field potential
MEA: multielectrode array
ms: milliseconds
nAChR: nicotininc Acetylcholine receptor
TBS: theta-burst stimulation
